# Knowledge Discovery in Ubiquitous and Personal Sleep Tracking: Scoping Review

**DOI:** 10.2196/42750

**Published:** 2023-06-28

**Authors:** Nhung Huyen Hoang, Zilu Liang

**Affiliations:** 1 Graduate School of Engineering Kyoto University of Advanced Science Kyoto Japan

**Keywords:** sleep tracking, knowledge discovery, data mining, personal informatics, self-experimentation, sleep health, scoping review, mobile phone

## Abstract

**Background:**

Over the past few decades, there has been a rapid increase in the number of wearable sleep trackers and mobile apps in the consumer market. Consumer sleep tracking technologies allow users to track sleep quality in naturalistic environments. In addition to tracking sleep per se, some sleep tracking technologies also support users in collecting information on their daily habits and sleep environments and reflecting on how those factors may contribute to sleep quality. However, the relationship between sleep and contextual factors may be too complex to be identified through visual inspection and reflection. Advanced analytical methods are needed to discover new insights into the rapidly growing volume of personal sleep tracking data.

**Objective:**

This review aimed to summarize and analyze the existing literature that applies formal analytical methods to discover insights in the context of personal informatics. Guided by the problem-constraints-system framework for literature review in computer science, we framed 4 main questions regarding general research trends, sleep quality metrics, contextual factors considered, knowledge discovery methods, significant findings, challenges, and opportunities of the interested topic.

**Methods:**

Web of Science, Scopus, ACM Digital Library, IEEE Xplore, ScienceDirect, Springer, Fitbit Research Library, and Fitabase were searched to identify publications that met the inclusion criteria. After full-text screening, 14 publications were included.

**Results:**

The research on knowledge discovery in sleep tracking is limited. More than half of the studies (8/14, 57%) were conducted in the United States, followed by Japan (3/14, 21%). Only a few of the publications (5/14, 36%) were journal articles, whereas the remaining were conference proceeding papers. The most used sleep metrics were subjective sleep quality (4/14, 29%), sleep efficiency (4/14, 29%), sleep onset latency (4/14, 29%), and time at lights off (3/14, 21%). Ratio parameters such as deep sleep ratio and rapid eye movement ratio were not used in any of the reviewed studies. A dominant number of the studies applied simple correlation analysis (3/14, 21%), regression analysis (3/14, 21%), and statistical tests or inferences (3/14, 21%) to discover the links between sleep and other aspects of life. Only a few studies used machine learning and data mining for sleep quality prediction (1/14, 7%) or anomaly detection (2/14, 14%). Exercise, digital device use, caffeine and alcohol consumption, places visited before sleep, and sleep environments were important contextual factors *substantially* correlated to various dimensions of sleep quality.

**Conclusions:**

This scoping review shows that knowledge discovery methods have great potential for extracting hidden insights from a flux of self-tracking data and are considered more effective than simple visual inspection. Future research should address the challenges related to collecting high-quality data, extracting hidden knowledge from data while accommodating within-individual and between-individual variations, and translating the discovered knowledge into actionable insights.

## Introduction

In tandem with the advent of consumer wearable technologies, there has been a growing interest in using consumer sleep tracking technologies for personal health management. Being aware of the importance of having a good night’s sleep, many individual users are routinely monitoring their sleep [1-3], and sleep tracking has been a popular topic, especially in the quantified-self community. Consumer sleep tracking technologies are largely divided into 2 types: smartphone dependent and smartphone independent. Smartphone-dependent sleep tracking technologies leverage the integrated sensors of a smartphone (eg, accelerometer, gyroscope, and microphone) to measure body movements and ambient sound, based on which a user’s sleep states can be estimated. Smartphone-independent sleep tracking technologies use independent hardware with multiple sensing modalities, such as accelerometers and photoplethysmography. These devices often come in the form of a wristband (eg, Fitbit [Fitbit Inc] and Apple Watch [Apple]), headband (eg, SleepSheperd [Sleep Shepherd] and Neuroon [Vandrico Inc]), or finger ring (eg, Oura [Oura Health Oy]), and they rely on proprietary sleep staging algorithms to calculate sleep metrics based on measurable physiological signals [4]. The accuracy of these consumer technologies has been significantly improved over the years. Recent models have proven to be reasonably accurate, especially in measuring the time of sleep onset and offset, total sleep duration, and sleep efficiency (SE) [5-7]. A recent study comparing 7 consumer sleep tracking devices with polysomnography (the gold standard of sleep measurement) demonstrated that their validity could outperform medical-grade actigraphy [6]. In the past decade, sleep tracking has become one of the most studied topics in the research field of personal informatics. At the intersection of ubiquitous computing, human-computer interaction, and sleep science, researchers from multiple disciplines have made joint efforts to investigate the validity of existing consumer sleep tracking devices [5-10], develop accurate sleep staging algorithms tailored to consumer sleep tracking devices [11-15], develop smartphone apps for visualizing personal sleep data [16-19], develop artificial intelligence–based sleep coaching systems that help people improve sleep hygiene [20], and understand the challenges for sleep tracking technologies to eventually improve sleep health [3,21-23]. At a higher level, sleep tracking studies have mostly been guided by general self-tracking frameworks, such as the lived informatics model [24] and the Prevetiver Health care on Individual Level framework [25]. Both frameworks emphasize the iterative exploration and analysis of the self-tracking data to gain insight and drive behavioral changes.

One of the known challenges in sleep tracking is how to empower layperson users to make sense of their sleep data and to identify lifestyle or environmental factors that they can modify for better sleep [22]. In the field of health informatics, health data analytics could be divided into multiple levels according to their analytical capabilities [26]. Depending on its outcomes, health analytics can be descriptive, diagnostic, predictive, or prescriptive in nature [27]. At the lowest level lies *descriptive analytics*, which answers the question, *what has happened?* This level of analytics describes data *as is* without applying complex calculations and exploration. Common techniques at this level, such as standard reports and alerts, focus on categorizing, characterizing, aggregating, and classifying data to understand the past and current states. Existing sleep tracking analytics is mostly centered on this level, which aims to help users gain a *nice-to-know* validation of their subjective perception of sleep. At the second level is *diagnostic analytics*, which focuses on possible antecedents and answers the question, *why did it happen?* This level of analytics requires extensive exploration and directed analysis and inference based on existing data to identify the potential problems and their probable causes. At the third level lies the *predictive analytics,* which focuses on the possible consequences and answers the question, *what is likely to happen next?* Sleep tracking technologies at this level should be able to predict users’ sleep quality in the near or far future by examining their historical self-tracking data, detecting patterns, and then leveraging the patterns to forecast. The highest level of analytics is *prescriptive analytics,* which answers the question, *what should be done about it?* It uses domain knowledge in medicine and health science in addition to data to generate recommendations for health interventions (eg, recommendations for better sleep).

Table 1 provides a mapping of the 4 levels of the analytics framework by Burke [26] for sleep tracking. The descriptive analytics would focus on answering questions such as “How many hours did I sleep last night?” “How many awakenings did I have last night?” and “What is the average deep sleep ratio during the past one month?” So far, sleep tracking has predominantly centered on such descriptive analytics, typically by visualizing data with charts and tables on a dashboard. This type of application could be meaningful in understanding users’ current sleep patterns. However, with a flux of multiple models of sensor data, simple data visualization may miss important patterns that are not easily observable through visual inspection. As the complexity of sleep tracking data increases, it becomes necessary to examine the data in a more structured manner using advanced analytics. For example, diagnostic analytics could help answer questions such as “Was my sleep normal?” or “Why did I have so many awakenings last night?” Predictive and prescriptive analytics could answer questions such as “How will my sleep quality be in five years if I keep going to bed at 2:00 am?” or “Will I sleep better tonight if I work out 10 minutes longer in the morning?” To achieve advanced analytics, it is necessary to combine different streams of contextual information into a sleep analysis. Although many consumers’ sleep tracking systems support the simultaneous collection of multiple streams of contextual information, these data are often visualized separately and rarely integrated with sleep analysis. Currently, there is a paucity of advanced analytics in sleep tracking research.

Knowledge discovery is the process of finding meaningful patterns from data, and data mining is a central step within a knowledge discovery process. Popular data mining techniques, such as association rules mining and anomaly detection, are widely used in many application domains to detect hidden patterns in large data sets [26]. In this paper, we present a scoping review on the application of knowledge discovery methods in sleep tracking. Such a review is useful for technical researchers interested in applying a wide range of machine learning and data mining techniques to the personal health domain as well as for sleep scientists who want to leverage the latest wearable technology combined with a data-driven approach for personalized and nonpharmaceutical interventions. Previous reviews on consumer sleep tracking technologies have dominantly focused on the utility and validity of these devices, especially in terms of their strengths and limitations relative to more widely accepted devices [4,28,29]. To the best of our knowledge, this scoping review is the first to focus on the advanced data analytics related to sleep tracking. An advanced data-driven approach has the potential to discover meaningful patterns or hidden correlations that could be used to guide behavioral change for better sleep. On the basis of the scoping review, we highlight the research opportunities for data-driven sleep computing.

**Table 1:** Level of analytics and its mapping to sleep tracking.

Analytics level	Questions answered	Mapping to sleep tracking
Descriptive	What has happened?	“How many hours did I sleep last night?”
Diagnostic	Why did it happen?	“Why did I have so many awakenings last night?”
Predictive	What is likely to happen next?	“How will my sleep quality be in five years if I keep going to bed at 2:00 am?”
Prescriptive	What should be done about it?	“Will I sleep better tonight if I work out 10 minutes longer in the morning?”

## Methods

### Research Questions

This scoping review was guided by the problem-constraints-system framework, which is widely used for conducting a literature review in computer science. The problem-constraints-system framework focuses on 3 different aspects of a research topic in computing research: a specific problem of interest (P); systems, applications, or algorithms (S) for tackling the problem; and constraints (C). After iterative brainstorming, we proposed the following 4 research questions (RQs) to anchor the entire review process:

- RQ1: What is the general research trend of knowledge discovery in sleep tracking?
- RQ2: What sleep quality metrics and contextual factors were considered, and how were they measured?
- RQ3: What knowledge discovery methods or algorithms were applied? What knowledge was discovered?
- RQ4: What challenges exist? What are the opportunities for future research?

### Search Strategy and Query String

In this review, we focused on the application of data mining to identify the relationships between sleep and contextual factors with consumer wearable devices. Therefore, search results must contain all 4 pertinent aspects: sleep metrics, contextual factors, wearable devices, and knowledge discovery. Automated searches were conducted in a number of databases, including the Web of Science, Scopus, ACM Digital Library, IEEE Xplore, ScienceDirect, Springer, Fitbit Research Library, and Fitabase. As our review was centered on the computing aspect rather than the clinical aspect of sleep tracking, we used databases that focus more on engineering and computer science publications, including the ACM Digital Library, IEEE Xplore, ScienceDirect, and Springer. PubMed was not used because we were not interested in cohort studies or medical assessments. We also included gray literature such as the Fitbit Research Library and Fitabase because of their relevance to our topics of interest. The query strings were slightly different for each database but always contained 4 main keyword combinations: “sleep,” “lifestyle” AND “contextual,” “data mining” OR “knowledge discovery,” and “wearable device.” Synonyms words and words of related concepts (eg, “self-tracking,” “sensors,” “machine learning,” “correlation,” and *“*statistics”) were also listed to avoid missing out on related publications. The search strategy varied for each search engine, as each of them had different rules and options. The query string was truncated in some databases (eg, Web of Science, ScienceDirect, and IEEE Xplore) either because long query strings resulted in many irrelevant entries or because of word limit. We included several types of publications, including journal articles, conference proceeding papers, workshop presentations, patents, and gray literature, to gain a broad scope of the research topic. Editorial articles, theses, and dissertations were also excluded.

### Study Selection

**Figure 1:**
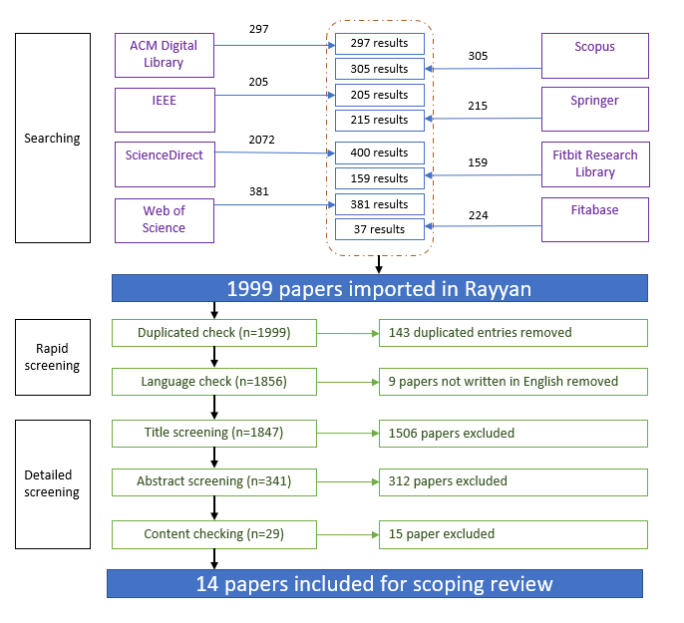
PRISMA (Preferred Reporting Items for Systematic Reviews and Meta-Analyses) flowchart of article screening process.

The study selection process is shown in the PRISMA (Preferred Reporting Items for Systematic Reviews and Meta-Analyses) flowchart in Figure 1. We applied the following exclusion criteria to filter out irrelevant papers retrieved from the databases: (1) studies not related to human sleep (eg, animal studies and sleep mode of sensor system), (2) clinical studies aiming at treating sleep disorders, (3) hypothesis-driven laboratory-based studies using invasive sleep tests (eg, polysomnography), (4) validation studies focusing on comparing wearable devices with medical devices, (5) studies focusing on estimating sleep architecture based on concurrent physiological signals, and (6) papers not written in English. We retrieved the returned entries from each database and imported them into the web-based review management software Rayyan (Rayyan). All databases provided tools to export the returned entries in a single file, except for the Fitbit Research Library and Fitabase. We exported only 400 of the 2072 papers from the ScienceDirect because the rest of the papers were not relevant to our review topic. For the ScienceDirect database, the returned items were first listed using the “relevant order” tool provided by the database. We used an a prior condition to include the first 400 items. For the remaining items, we separated them into groups of 200 items and randomly checked 50 items in each group. We found that from the 400th item onward, the studies were off the topic according to our exclusion criteria. Thus, we eliminated these items because they did not help address our central RQs. For example, the keyword “contextual” led to the retrieval of papers in civil engineering on checking bedroom quality with air condition, ambient light, and noise exposure. Those were excluded based on the exclusion criterion 1. The keyword “wearable device” led to the retrieval of papers in electrical and mechanical engineering on hardware design for sensors, batteries, and ergonomic design. Those were excluded based on the exclusion criterion 4. For the Fitbit Research Library, 2 papers were missing because they were either retracted or no longer available. Entries in Fitabase were screened for duplicity before manually adding to Rayyan because Fitabase does not support automatic export. As many of the Fitabase entries were duplicates of publications from other databases, only 37 papers were added to Rayyan.

In total, 1999 papers were imported to Rayyan. Screening for duplication and written language was performed automatically in Rayyan. After the rapid screening step, the first author performed title and abstract screening and suggested the inclusion of 341 papers. The second author repeated the title and abstract screening of the 341 papers while paying special attention to the exclusion criteria 3 and 5. Any conflicts were resolved through discussion with the first author. After the screening and eligibility checks, 29 papers remained. Both authors read the full text of the 29 papers in detail and finally selected 14 papers for the scoping review.

## Results

### General Research Trend (RQ1)

Ubiquitous and personal sleep tracking has become an active research area since approximately 2011, when a few pioneer studies were published in the human-computer interaction community [17,18,30]. Since then, a large number of studies on sleep tracking have been published, but most of them are dominantly centered on the validation of existing sleep tracking devices or systems [5-10], on the development of new sleep staging algorithms [11-15], or on the investigation into users’ experience with the technologies [3,21-23]. In contrast, studies that focus on knowledge discovery in sleep tracking data are scarce and ad hoc. The screening process identified only 14 relevant studies, of which more than half (8/14, 57%) were conducted in the United States, followed by Japan (3/14, 21%). The other publications were from China, Korea, Finland, and Australia. Only 5 (36%) of the 14 publications were journal articles, and the rest were conference proceeding papers. Chronologically, studies by Jayarajah et al [31] and Gelman and Hill [32] were one of the earliest studies in this field. The authors developed a binary tree model to predict good and poor sleep based on app use activity and social time during the day. Since 2015, the topic of knowledge discovery in sleep tracking has begun to attract more attention along with the advances in consumer sleep tracking technologies. As a result, the number of publications has increased slightly in the subsequent years, but the total amount is still limited.

A common objective of these studies was to help users gain insights into how their sleep quality was associated with other aspects of their daily lives. Some of the specific motivations are as follows:

- Identify aspects of daily life that demonstrate significant associations with personal sleep quality from self-tracking data [16,20,33]
- Highlight the potential for sleep metrics from wearable devices to provide novel insights into data generated from a large cohort [34-36]
- Detect aberrant sleep patterns or typical events during sleep by considering individuals’ sleep baselines [37,38]
- Guide users in designing self-experiments to identify personal modifiable lifestyle factors for better sleep health [20]
- Develop a recommender system that provides both general and personalized recommendations for better sleep health [20]

The studies reviewed in this paper demonstrated a tendency to analyze sleep tracking data along with a flux of contextual factors. These factors were used as independent variables for predicting sleep quality and, to a lesser degree, for identifying antecedent events that affect sleep quality. In the scheme of traditional sleep science studies, only a limited number of independent variables were considered, and the confounding effect of noninterested factors needed to be controlled through a rigid experiment design. In comparison, data collection experiments in ubiquitous sleep tracking studies are often conducted in a naturalistic environment, making it challenging to control for confounding factors. Therefore, advanced data analysis techniques are required to control the effects of confounding factors during the analysis. Another line of research effort is the personalized detection of aberrant sleep. Although sleep quality assessment may sound straightforward using clinical standards [39], it remains challenging if personal differences in sleep needs should be taken into consideration. Studies in this direction are limited, and we identified only 2 relevant studies [37,40].

### Quantification and Measurement of Sleep and Contextual Factors (RQ2)

Human sleep can be quantified along multiple dimensions, such as sleep duration, sleep continuity, sleep timing, and subjective perception of the sleep event [41]. We found that not all studies used the same set of metrics to characterize sleep quality. Even the same sleep metric may be capsulated in different terminologies across studies. To facilitate cross-study comparisons, we mapped the sleep metrics in each study to standard clinical terms, whenever possible. Original sleep metrics that have no corresponding clinical terms were simply left as they were. As presented in Table 2, the most used sleep metrics among the reviewed studies were subjective sleep quality (4 studies), sleep efficiency (SE, 4 studies), sleep onset latency (SOL; 4 studies), and time at lights off (3 studies). Ratio parameters such as deep sleep ratio and rapid eye movement ratio were not used in any of the studies. Moreover, the cutoff between good and poor sleep varied from study to study. A few studies have adopted clinical cutoffs, particularly for the Pittsburgh Sleep Quality Index (5) [31], SE (85%) [42], wake after sleep onset (30 minutes) [16], and total sleep time (7-9 hours) [16]. The rest chose to use heuristic cutoffs that did not comply with the clinical guidelines.

**Table 2:** Sleep quality metrics, measurement methods, and cutoff of good or poor sleep.

Clinical term, original term, and measurement method			Cutoff
**Subjective sleep quality**			
	Pittsburgh Sleep Quality Index [31,38]	Good: ≤5 and poor: >5 [31]; good: ≤7 and poor: >7 [38]	
	Sleep rating (SleepAsAndroid app) [20]	N/A^a^	
	Leeds Sleep Evaluation Questionnaire [19]	N/A	
	Sleep rating (SleepApp) [19]	N/A	
**SE^b^**			
	SE (Fitbit) [33]	Good: ≥95% and poor: <95%	
	Efficiency (MS Band) [43]	N/A	
	SE (Polar) [40]	Good: >mean (SD) and poor: <mean (SD)	
	SE (Garmin) [42]	Good: ≥85% and poor: <85%	
**SOL^c^ (minutes)**			
	Minutes to fall asleep (Fitbit) [16]	N/A	
	SOL (SleepAsAndroid app) [20]	N/A	
	Sleep latency (Garmin) [42]	Good: ≤15, average: 15-30, and poor: >30	
	Time to fall asleep (MS Band) [43]	N/A	
**Time at lights off**			
	Bedtime (Fitbit) [35]	Normal bedtime: median of a participant’s bedtimes; deviation categories were 1-30 minutes, 30-60 minutes, 1-2 hours, 2-3 hours, and ≥3 hours	
	Bedtime (Fitbit) [37]	N/A	
	Bedtime as estimated by the time of the last network signal [36]	N/A	
**Wake after sleep onset (minutes)**			
	Awake minutes (Garmin) [42]	Good: ≤20 and poor: >20	
	Minutes awake (Fitbit) [16]	Good: ≤30 and poor: >30	
**Number of awakenings >5 minutes**			
	Awakenings >5 minutes (Garmin) [42]	Good: ≤1 and poor: >1	
	Number of wakeups (MS Band) [43]	N/A	
**Time in bed (minutes)**			
	Time in bed (MS Band) [34]	N/A	
**Total sleep time (minutes)**			
	Minutes asleep (Fitbit) [16]	Good: 420-540 and poor: <420 or >540	
**Original metrics (no corresponding medical term)**			
	Sleep ratio=the ratio of the sleep minutes with normal heart rate versus total sleep time (Fitbit) [44]	Good: >90%, normal: 60%-90%, and bad: <60%	
	Number of awakenings per hour [20]	N/A	
	Awakening count including restlessness (Fitbit) [16]	N/A	
	Permutation entropy of Fitbit measured sleep state time series [37]	N/A	

^a^N/A: not applicable.

^b^SE: sleep efficiency.

^c^SOL: sleep onset latency.

Sleep quality metrics were measured using 3 methods: questionnaire based, app based, and wearable based. The Pittsburgh Sleep Quality Index was the most widely used questionnaire to measure the subjective perception of sleep duration, continuity, efficiency, and satisfaction [31,38]. The SleepAsAndroid app was used to collect sleep data based on user movement patterns in the study by Daskalova et al [20], and the SleepApp was used to collect users’ subjective sleep quality ratings in the study by Ravichandran et al [19]. Although sleep tracking apps such as SleepAsAndroid were widely downloaded and used by millions of users, their ability to distinguish between quiet awakenings, deep sleep, or empty beds was still limited [20]. In a related vein, studies by Jayarajah et al [31] and Faust et al [36] have defined sleep time as the longest period during which there was no activity on users’ smartphones. However, the authors acknowledged that this method was not reliable because not everyone had the habit of using smartphones until they fell asleep. Most studies (9/14, 64%) used commercial wearable devices such as Fitbit [16,33,35,37,44], Microsoft Band (MS Band) [34,43], Garmin [42], and Polar [40]. Although all 3 methods are noninvasive, easy to use, and allow longitudinal collection of sleep data, each method has some limitations. The questionnaire-based method is subject to memory recall bias. Sleep data collected by sleep tracking apps and wearable sleep trackers provide an objective description of sleep quality and sleep structure. However, they may also be prone to measurement errors because of hardware and software limitations [7,28]. They also require users to place the smartphone nearby or to wear the device continuously, which may cause discomfort during long-term use. Moreover, despite being small and convenient, consumer wearable trackers cannot provide hypnogram information that is as detailed as medical devices.

Along with sleep quality metrics, researchers have considered a wide range of contextual factors in their studies. As presented in Table 3, the contextual factors of interest include the sleep environment [16,20,42], daily activities [16,19,20,31,36,37,40,42,43], physiological states [16,35,36,43,44], and mental states [16,19]. It was a common practice to collect demographic information (eg, age, gender, and BMI) and medical history using questionnaires at the beginning of a sleep tracking study [16,35,36,43,44]. Other contextual factors were recorded during the data collection experiments. Researchers have been curious about how web activities of university students could be coupled with their sleep quality [31,34,36]. These studies developed their own tools to track users’ web-based behavior and linked them to sleep quality metrics. Using consumer wearable trackers such as Fitbit, MS Band, and Garmin, researchers were able to expand their list of factors to include, for example, bedtime, steps, distance, hours of exercise, and calories burned [16,19,20,31,36,37,40,42,43]. Biosignals such as heart rate during sleep and daytime activities were included in the studies by Faust et al [35], Farajtabar et al [43], and Choi et al [44]. Several studies have also manually collected input features such as coffee, alcohol, mood, and stress [16,19,20]. These factors may have a significant effect on the circadian cycle of hormone secretion and thus may provide useful information for sleep quality prediction. However, collecting these data are nontrivial, as users tend to forget to log the data on a daily basis. How to collect these data more efficiently and how to reduce the risk of missing data remain challenging.

**Table 3:** Contextual factors and measurement methods.

Category and contextual factor			Data collection method
**Physiological factors**			
	Age	Self-report [43,44]	
	Sex	Self-report [36,43]	
	Body weight	Fitbit [16] and self-report [43]	
	BMI	Self-report [44]	
	Body temperature	Diary [16]	
	Heart rate	Fitbit [35,44] and MS Band [43]	
	Menstrual cycle	Diary [16]	
	Calorie in and out	Fitbit [16] and MS Band [43]	
	Activity calorie	Fitbit [16]	
**Psychological factors**			
	Stress	Diary [16]	
	Mood	Diary [16] and SleepApp [19]	
	Tiredness	Diary [16]	
	Dream	Diary [16]	
	Sleep quality the previous night	SleepAsAndroid [20] and MS Band [43]	
	Cognitive performance	Keystroke time [34] and click time [34]	
**Behavioral factors**			
	Steps	Fitbit [16,37] and MS Band [43]	
	Distance walked	Fitbit [37]	
	Active time	Fitbit [16,37]	
	Exercise	SleepAsAndroid [20], MS Band [43], Polar [40], Garmin [42], and SleepApp [19]	
	Coffee	Diary [16], SleepAsAndroid [20], and SleepApp [19]	
	Alcohol	Diary [16], SleepAsAndroid [20], and SleepApp [19]	
	Tobacco	Self-report [44] and SleepApp [19]	
	Electronic device use	App use time (total and different app categories) [31], diary [16], Bing search logs [43], SleepApp [19], and campus network [36]	
	Nap	Diary [16], SleepAsAndroid [20], and SleepApp [19]	
	Location	Campus Wi-Fi [31] and Cortana [43]	
	Social activity	GruMon (location estimation based on Wi-Fi signals) [31], diary [16], and Twitter [43]	
	Mealtime	Diary [16], smartphone camera [42], SleepApp [19], and campus smart card [36]	
	Waketime	SleepAsAndroid [20]	
	Bedtime	Fitbit [37], IoT^a^ sensor [42], and SleepApp [19]	
**Environmental factors**			
	Ambient temperature	Diary [16] and IoT sensor [42]	
	Ambient humidity	Diary [16] and IoT sensor [42]	
	Ambient light	Diary [16] and SleepAsAndroid [20]	
	Ambient noise	SleepAsAndroid [20]	
	Day of week	MS Band [43]	

^a^IoT: internet of things.

We found that 13 (93%) of the 14 reviewed studies conducted their own data collection experiments [16,31,33,37,38,43,44] in free-living conditions. In these studies, sleep data were recorded in participants’ usual sleep environments (eg, homes and caregiving facilities), whereas contextual factors were recorded while participants were at schools, universities, workplaces, or sports centers. In contrast, only 1 study used an existing data set [35], which can be accessed over the web [45]. Data sharing is not yet a common practice in the field, and the number of public data sets is limited.

### Knowledge Discovery in Sleep Tracking (RQ3)

#### Data Preprocessing

All the reviewed studies used data sets that were collected in free-living environments. Data collection “in the wild” increases the ecological validity of the studies but at the sacrifice of data quality. The first step in the knowledge discovery process is to deal with missing, wrong, and duplicate data. Although there are many methods for data cleaning in the literature, the methods adopted in the reviewed publications are extremely simple and straightforward. Most of the studies (5/14, 36%) simply excluded records that contain missing values (eg, sleep logs with missing fields or sleep duration of 0 minutes) [16,19,37] or excluded users who do not contribute sufficient data (eg, fewer than 30 sleep records) [35,36]. One study excluded a certain data source (eg, search engine interactions originating from mobile devices) to avoid causing distortion to the data distribution [34].

Similarly, data out of logical ranges were removed. Users aged <10 years or >100 years, with weight <22.7 kg or >112.5 kg, or with height <127 cm or >457.2 cm were excluded in the study by Farajtabar et al [43]. Extremely short (<0.5 or 4 hours) and long (>12 hours) sleep records were removed in the studies by Althoff et al [34] and Farajtabar et al [43]. Sleep entries with bedtime between 7:00 AM and 7:00 PM were removed in the study by Liang et al [33]. Exercise time is another criterion for filtering out potentially erroneous data records. For example, exercise events <5 or >180 minutes were removed in the study by Farajtabar et al [43]. Exercises with calorie consumption per hour <50 or >2000 calories or with a duration of <10 minutes were excluded in the study by Liu et al [40]. In addition, data records with steps <1000 and those with a sedentary time of 0 minute were removed in the study by Liang et al [16].

Time stamps are another focus in data preprocessing. The data collection in the study by Ravichandran et al [19] primarily relied on users’ manual input in SleepApp and thus had a higher risk of human errors. Consequently, all logs with aberrant timestamps were removed. In addition, 12 hours were added to or subtracted from the recorded bedtime or wake-up time where the users might have forgotten to toggle the AM and PM switch on the app. Dealing with timestamps involves not only data cleaning but also temporal matching among multiple data sources [42] as well as data type conversion (eg, 18:30 to 1830) [16,37].

Other types of data preprocessing included selecting users with larger variations in sleep and exercise [38], removing redundant entries [19], and resampling the raw data (eg, the photoplethysmography-derived heart rate time series was aggregated every 3 minutes to achieve a constant sampling rate [38]).

Some knowledge discovery processes that rely on machine learning or data mining techniques may require a feature engineering process instead of directly using the cleaned data as input. For example, several studies have involved the construction of secondary features from the cleaned data [37,38,40]. Features were normalized to have a mean and SD equal to 0 and 1 [42] or normalized over another feature (eg, exercise intensity features were normalized by dividing the basal metabolic rate [38]). Dimension reduction (eg, principal component analysis) was applied to reduce the number of input features to avoid the adverse effect of the “curse of dimension” [31].

#### Data Mining

The selection of the data mining method depends on the purpose of the studies and, to a lesser degree, on the size of the available data set. Table 4 provides a summary of the data mining methods and the specific techniques or algorithms used in the reviewed studies. We also listed the independent variables (or input) and dependent variables (or output) of the constructed models. Correlation analysis, regression analysis, and rule induction are the most used methods for finding meaningful associations between contextual factors and sleep quality metrics. In total, 3 correlation analysis techniques, Pearson correlation, Spearman correlation, and repeated measure correlation, were applied to examine the strength of the pairwise linear relationships between sleep and contextual factors [16,19,20]. Similarly, various regression analysis methods have been used, ranging from simple linear regression to linear mixed effects regression to piecewise fixed effects regression [34,35,43]. Least square estimation was the most popular technique for parameter estimation in regression analysis and was used in the studies by Althoff et al [34] and Farajtabar et al [43]. The study by Faust et al [35] provided no information but is highly likely to use the same technique. It is worth noting that the Pearson correlation coefficient is equivalent to the standardized slope of a simple linear regression line.

**Table 4:** Summary of the data mining methods used in the reviewed studies.

Data mining method and techniques or algorithms			Data size		Independent variable		Dependent variable
**Correlation analysis**							
	Pearson correlation [20]	Preliminary study: 24 users over 20 days; final study: 19 users over 21 days		TST^a^ and contextual factors		SOL^b^, NAWK^c^, and sleep rating	
	Spearman correlation [16]	12 users over 2 weeks		Contextual factors		TST, WASO^d^, NAWK, SOL, and SE^e^	
	Repeated measure correlation [19]	10 users over 2 weeks		Bedtime, TIB^f^, and contextual factors		SE, SOL, NAWK, restlessness, TIB, and LSEQ^g^	
**Regression analysis**							
	Piecewise fixed effects regression [34]	31,793 users over 18 months; all American users		Time of day, time after waking up, and sleep duration		Cognitive performance	
	Simple linear regression [43]	Approximately 20,000 users over 4 months		Contextual factors		SOL, NAWK, and SE	
	Linear mixed effects model [35]	557 users over 1 year		Bedtime regularity		Resting heart rate	
**Rule induction**							
	A priori algorithm [33]	1 user over 180 days; 4 users over 2 weeks		Contextual factors		SE	
	Learn from Examples using Rough Sets [44]	280 users over 1 month; only the data of males were used		Contextual factors		Sleep ratio	
	Event mining (+causal inference) [42]	1 user over 800 days		Contextual factors		SOL, WASO, NAWK, and SE	
**Causal inference**							
	Stratified propensity score analysis [43]	Approximately 20,000 users over 4 months		Contextual factors		SOL, NAWK, and SE	
	Bayesian network analysis [36]	5200 users over 6 months		Contextual factors and bedtime		Contextual factors and bedtime	
**Time series analysis**							
	Anomaly detection [37]	1 user over 35 days		Fitbit measured intraday time series, TST, WASO, NAWK, and bedtime		Permutation entropy of sleep time series	
	SAX^h^-based motif matching and principle optimization [38]	100 users over 10 weeks		Heart rate time series data		PSQI^i^	
**Statistical test**							
	Unpaired 2-samples Wilcoxon test [40]	271 users over 8 months		Contextual factors		Statistical differences between good and poor sleep	
**Decision tree**							
	J4.8 Classifier [31]	400 users over 15 months		Contextual factors		PSQI	

^a^TST: total sleep time.

^b^SOL: sleep onset latency.

^c^NAWK: number of awakenings.

^d^WASO: wake after sleep onset.

^e^SE: sleep efficiency.

^f^TIB: time in bed.

^g^LSEQ: Leeds Sleep Evaluation Questionnaire.

^h^SAX: Symbolic Aggregate Approximation.

^i^PSQI: Pittsburgh Sleep Quality Index.

Although correlation analysis and regression analysis (except piecewise constant approximation) capture the relationships between sleep and contextual factors in the entire sampling range, the piecewise regression in the study by Althoff et al [34] shared some resemblance with rule induction methods that capture the relationship between sleep and contextual factors within a constrained range. However, in contrast to piecewise regression, which quantifies the covariance between 2 variables in each partitioned segment, rule induction methods focus on extracting frequent patterns in the data sets that characterize the co-occurrence of 2 variables when their values fall into the corresponding ranges specified in a rule. Moreover, rule induction methods are usually robust to missing data. As the rule induction methods were originally developed to analyze categorical data, numerical data need to be converted to categorical data through a discretization step before rule induction methods can be applied. There are 4 discretization methods used in the reviewed studies: equal size discretization [33,44], equal frequency discretization [33], k-means clustering discretization [33], and discretization with heuristically defined cutoffs [42]. Association rules mining is a popular rule induction method that has been widely used in traditional medical and health informatics applications; however, it has only been used in 1 study among all the studies we reviewed [33]. In that study, the a priori algorithm was applied for rule induction. The quality of the induced association rules was validated by higher local correlation coefficients (ie, the Pearson correlation coefficient when the variables fall into the ranges specified in a rule) than the global correlation coefficients (ie, the Pearson correlation coefficient between 2 variables within the entire sampling range). To better handle the potential inconsistency (eg, conflicting records) in the data set, Rock-Hyun et al [44] applied another rule induction algorithm named learning from examples using rough sets. The global covering algorithm computes the lower and upper approximations of all the target sleep quality metrics (eg, sleep ratio=“good”) if the input data set contains conflicting records. The quality of the induced rules was assessed based on the predictive accuracy of the target sleep metrics. Although association rules mining and learning from examples using rough sets capture only the parallel co-occurrence of 2 items (ie, when the values of 2 variables fall into the corresponding ranges specified by a rule), event mining can also capture the co-occurrence of 2 items with a time lag (ie, the temporal sequence when the 2 items occur) [46]. To a certain degree, event mining resembles sequential pattern mining [47]; however, this characteristic was not used in the study by Upadhyay et al [42].

Causal inference is a powerful approach to reduce potential bias in the identified relationships between sleep and contextual factors because of observed confounding factors. This method is likely to outperform simple correlation analysis or rule induction–based methods. In the study by Farajtabar et al [43], stratified propensity score analysis was performed to isolate the effects of potential confounding factors. A similar technique was used in the study by Upadhyay [42] to enhance the quality of the induced rules by accommodating confounding factors. In addition, the Bayesian network was applied to explore the relationship between sleep schedules and behavioral factors [36].

Statistical tests and decision trees were also used in the existing literature, but only in 1 study each. The unpaired 2-samples Wilcoxon test was applied to identify significant differences in a set of selected contextual factors between good and poor sleepers [40]. Despite its simplicity, this method does not generate quantitative relationships between the contextual factors and sleep quality. In contrast, decision trees were used in the study by Jayarajah et al [31] to predict sleep quality using contextual factors as input features.

In addition to the abovementioned methods, time series analysis was also used but only in 2 studies [37,38]. Dimension reduction and anomaly detection were combined to identify aberrant sleep recordings while counting in personal sleep baseline in the study by Liang et al [37]. Feng and Narayanan [38] introduced a method to discover motifs in heart rate time series, which were signal patterns that appeared most frequently during sleep [38]. They then used these motifs as features to predict sleep quality. In contrast, although the study by Upadhyay et al [42] adopted a streaming data perspective, it did not incorporate any formal time series analysis technique [39].

#### Knowledge Discovered

The knowledge discovery process in the reviewed studies identified interesting associations both at the cohort level and the individual level. First, significant associations were found among the sleep quality metrics. Late bedtime was associated with a higher permutation entropy of the Fitbit measured sleep time series (indicating a higher chance of aberrancy) [37]. Bedtime deviation was correlated to longer SOL [19]. In addition, sleep duration was positively associated with subjective sleep satisfaction [19,20].

Regarding the relationship between sleep quality and contextual factors, exercise was the most identified association factor [33,40], but the relationship between exercise and sleep was complex [40,43]. First, not all exercise features have a predictive power of sleep quality. For example, Liu et al [40] found that exercise duration, relative calories consumption, and exercise timing could be used as predictors of sleep quality, but exercise intensity was not significantly associated with sleep quality. Second, exercise may be positively associated with some sleep quality metrics but negatively associated with others. For example, exercise before bed may be linked to shorter SOL and higher SE [43], and exercise seems to improve SOL the most among all the sleep quality metrics [42]. However, the results diverged as different types of exercises were considered. Taking more steps meant fewer awakenings, whereas running and burning more calories were correlated with more awakenings [43]. Moreover, longer exercise duration (eg, >100 minutes) may be associated with good sleep for some users but poor sleep for others [40]. Furthermore, confounding factors may modulate the relationship between sleep and exercise. With causal inference, it was found that pleasant ambient temperature at bedtime significantly strengthened the relationship between exercise and sleep, whereas having a poor sleep the previous night detracted from the beneficial effects of exercise [42].

Digital device use is another important factor that correlates with sleep quality and sleep schedule. No web searches before bed correlated with shorter SOL and higher SE, whereas web searches before bed correlated with more awakenings [43]. The association between app use and sleep quality was modulated by the frequency and timing of use [31]. In particular, low social app use was associated with good sleep, and more app use correlated with good sleep if used for >4 hours before sleep. Reading and gaming app use within 1 hour before bedtime correlated with poor sleep. A strong connection between internet surfing habits and bedtime was identified by Guo et al [36]. Video lovers tended to go to bed later than game fans. Going to bed late, in turn, has negative consequences. Deviations from usual bedtime may result in a higher resting heart rate during sleep [35]. Students who went to bed late were more likely to have a poor academic performance [36]. Late bedtime (in relation to one’s circadian cycle) reduced cognitive performance the next day, whereas early bedtime did not have the same negative effect [34]. In contrast, having a sufficient sleep was important for maintaining normal cognitive performance [34].

As expected, caffeine and alcohol consumption were significant association factors. Consuming caffeine late in the day was a universal negative factor in subjective sleep ratings [20]. Alcohol consumption was positively correlated with SE and wake-up freshness and was negatively correlated with wake after sleep onset and number of awakenings for some users but not all users [16].

Not just personal activities or substance consumption associated with sleep, but places visited before bedtime and social life also play a role. One study found that students who spent more time with friends had better sleep quality than those who stayed alone on campus most of the time [31]. In addition, if students spent most of their time outside campus, good quality sleep was found for those who spent <15% of their time being alone. Another study tracked users’ location during the day and stated that users took longer to fall asleep if they visited food-related or bank-related locations close to bedtime [43]. In addition, sleep quality may vary depending on the environment in which sleep takes place. The most common relationship identified for many users by Daskalova et al [20] was the pair of noisiness and number of awakenings [16]. Temperature was positively associated with all sleep metrics except for SOL [42].

### Challenges and Opportunities (RQ4)

Knowledge discovery in sleep tracking is the process of extracting nonobvious hidden knowledge from self-tracking sleep data and other available contextual information. Preparing a data set of a sufficient size is the first step in this process. Almost all the reviewed studies (13/14, 93%) conducted original data collection experiments using noninvasive wearable and mobile sensors. The existing literature highlighted several challenges of data collection in sleep tracking. First, the absence of an objective, quantifiable, and universal definition of good sleep places a big challenge in annotating the collected sleep data [16,19]. Without a well-annotated data set, it is not feasible to apply supervised data mining techniques, and the absence of ground truth impedes the unbiased evaluation of the knowledge discovery process. Second, some contextual factors are considered difficult to quantify. These factors include digital device use, caffeine and alcohol consumption, and social interaction [16], to name but a few. The timing of data logging may also influence the results [20]. For example, users were advised to avoid using digital devices 2 hours before bedtime but had to log on to their smartphones to submit daily data at the end of the day. Third, existing passive sensing methods may have strong limitations [34]. For example, some studies (2/14, 14%) assume that users check their smartphone right before bedtime and immediately after waking up [31,36] and thus may miss out on users who have no such habits. Consumer wearables may have limited accuracy in measuring sleep stages and other factors [7], but the issue of data quality was not considered in the reviewed studies [35].

Moreover, interpreting the knowledge discovery outcome is not always straightforward. Correlation analysis essentially captures the covariance of 2 variables. Users with regular sleep and daily life routines may end up with no significant correlations found because of the lack of variability in their data. However, users may misinterpret this as having no relationship [16]. Rule induction methods usually generate a large number of rules, but not all of them are useful. Long rules with too many factors in the antecedent, despite of being explainable, provide no actionable insights because of their complexity (eg, “IF 17.85 < BMI < 25.21 AND Smoking is Yes AND 61.81 < Normal_Avg_HR < 79.0 AND 0 < Normal_Awake <19.0 AND 1.5 < Normal_Really_Awake < 24.0 AND 1.5 < High_Asleep < 606.5 AND 1.5 < High_Awake < 155.0 AND 0.5 < High_Really_Awake < 175.5 THEN Sleep Quality Status is ‘Bad’ with support 8”) [44]. In contrast, short association rules may be more comprehensible (eg, “minutes very active={33; 38}=> good sleep or steps={18,658; 20,263}=> good sleep” [33]). However, heuristic discretization without a semantic meaning may impede understandability [20].

Despite the challenges, the reviewed studies highlighted several opportunities for future research. In total, 3 studies suggested considering more contextual factors in addition to the ones already studied, such as emotion, diet, productivity, and chronotypes [31,34,36,42]. Acknowledging that the correlations at a cohort level may be weak [31], argues for an individual-centric approach to identifying the most important contextual factors for each user. Along the same line [43], it was pointed out that building predictive models within similar user groups is more practical. They proposed a hierarchical modeling scheme with a top layer containing population parameters and lower layers personalized to individual users. Similar user profiling and segmented modeling proposals were presented in the studies by Liang et al [33] and Farajtabar et al [43].

## Discussion

### Principal Findings

Sleep tracking using consumer wearable devices and mobile apps has attracted remarkable attention from the research community. However, sleep tracking studies have focused on developing sleep tracking technologies for accurately measuring sleep per se, and little attention has been directed to the extraction of patterns and insights from these data. To the best of our knowledge, this scoping review is the first to map the existing literature from a knowledge discovery perspective in sleep tracking.

Our analysis results showed that the number of publications on the topic of interest has slightly increased over the years but is still low, probably because the data-driven scheme has not been fully embraced in personal informatics. Nonetheless, we found that the existing literature covered all 4 levels of analytics, as presented in Table 1. Most of the 14 studies that we reviewed applied simple correlation analysis, regression analysis, and rule induction methods to discover the associations between sleep and other aspects of life. Although most consumer sleep tracking technologies allow users to visually inspect their sleep data (which is descriptive in nature), the reviewed studies demonstrated the feasibility of diagnostic analysis with a flux of sleep and contextual data. Although correlation does not necessarily indicate causality, a combination of association analysis and causal inference—as was done in the study by Upadhyay et al [42]—may help users narrow down the scope of possible modifiable factors that are likely to affect their sleep quality. Machine learning and data mining techniques were only used in a few studies for anomaly detection (which is diagnostic) [37] or sleep quality prediction (which is predictive) [31,43]. In total, 2 studies developed computational models to generate personalized recommendations for better sleep and showed promise in prescriptive analysis of sleep tracking [20,42]. The most used sleep metrics among the reviewed studies were subjective sleep quality, SE, SOL, and time at lights off. Exercise, digital device use, places visited during the day and before bedtime, and sleep environment are the major factors that significantly correlate with various dimensions of sleep quality.

Taken together, there are a few key challenges that are relevant to the findings. On the one hand, it is nontrivial to collect high-quality data in naturalistic settings. Challenges include how to motivate users to overcome tracking fatigue, how to enhance the reliability of consumer wearables and apps, and how to quantify and automate the collection of contextual information to represent the current challenges surrounding the collection of sleep tracking data sets. On the other hand, how to extract hidden knowledge from data, how to accommodate commonness and individuality, and how to interpret data mining results are topics for future studies.

### Nuance in Handling Within-Individual Variation

The selection of appropriate data mining methods relies on a correct understanding of the nature of the sleep tracking data set. Researchers often conduct longitudinal data collection experiments that involve the collection of multiple measurements of the same variables (eg, sleep quality, exercise, and ambient light) from each individual user. The data form a hierarchical or clustered structure when aggregated at the cohort level. Caution must be exercised when applying traditional analytic and modeling techniques developed for single-level data, as hierarchical data are likely to violate the assumption of independent errors of those techniques. In particular, although a hierarchical data set offers the benefit of a larger amount of data, the within-individual variation at the individual level needs to be addressed carefully through multilevel analysis and modeling.

In a multilevel analysis framework, the repeated measures are clustered within the level of an individual, and each individual is treated as a cluster unit. Depending on whether the analysis of one cluster involves pooling the data of other clusters, there are 3 approaches to analyzing a hierarchical data set: complete pooling, no pooling, and partial pooling. Complete pooling completely ignores the variation between individual users and treats all samples as being drawn from the same population. A dominant portion of the studies reviewed in this work [16,31,33,34,36,38,40,43,44] adopted this approach. Nonetheless, this approach is undesirable, as it violates the assumption of independence. The results could have been distorted when the between-individual variation was high. At the other end of the spectrum lies the no pooling approach, where the analysis of the relationship between sleep and contextual factors was performed only on the data of each individual user without considering data from other users. Daskalova et al [20] Upadhyay et al [42] embraced an N-of-1 design and correspondingly adopted the no pooling approach to analyze the collected data. At the surface, this approach is plausible for fully handling the within-individual variation. However, it bears the risk of overstating the variation between individual users because of potential overfitting when the number of samples from individual users is small. Partial pooling or multilevel modeling compromises between pooled and unpooled estimates, with the relative weights of pooling determined by the sample size of each individual user and the variation within and between individuals. Multilevel modeling automatically adjusts the degree of pooling with a “soft constraint,” which ensures strong pooling for users with fewer records and weak pooling for users with abundant records in the data set [32]. We found that only Ravichandran et al [19] and Faust et al [35] used the multilevel modeling approach and explicitly considered the within-individual variation.

Most reviewed studies (8/14, 57%) seem to have relied on the undue assumption of an independent and identically distributed data set. As a result, some studies (2/14, 14%) found mixed or even conflicting results on the relationships between sleep and contextual factors at the cohort level [40,44] and, consequently, generated no valuable insights. Studies using an N-of-1 design are interesting exceptions. In these studies, analysis was conducted on each user’s data, thus eliminating the effect at the cohort level. However, the robustness and generalizability of the findings are questionable. Even in studies with an N-of-1 design, it may still be helpful to partially pool some samples from the population to increase the reliability of the model parameter estimates. As such, Gelman et al [48] suggested always using multilevel modeling (ie, “random effects”) as a rule of thumb, for example, linear mixed effect model over simple linear regression model and generalized linear mixed model trees [49] over the J4.8 classifier.

### Limitations of the Study

There is room for improvement in several aspects of this study. First, because of the limitations inherent in scoping reviews, this study is exploratory and primarily qualitative in nature. Limited by the review methodology, we were unable to generate a quantitative “summary of findings, ” as required for systematic reviews or meta-analyses. Second, our method is nonstandard in a sense that we performed prescreening on the items identified in the ScienceDirect database before importing all entries into Rayyan. Although we made an effort to ensure that the removed items were not relevant, we cannot rule out the possibility of missing publications that should have been included. Third, we did not conduct critical appraisal on the quality of the selected papers or perform a risk of bias assessment, which may have led to potential bias in the selection and interpretation of the papers. Despite these limitations, this review provides a well-scoped summary of existing research and could lay the groundwork for future systematic reviews. The research gaps that we identified can be used to inform future research agendas.

### Conclusions

This scoping review built an understanding of the scope and nature of existing literature on knowledge discovery in ubiquitous and personal sleep tracking. To the best of our knowledge, this is the first review that exclusively focused on the knowledge discovery aspect of self-tracking in the realm of sleep health. In total, 14 studies were included in the review based on the exclusion criteria. We found that the existing literature covered all 4 levels of the analytics framework in health informatics. However, half (7/14, 50%) of the studies have only applied simple correlation analysis and regression analysis, aiming to discover significant associations between sleep and available contextual information. Machine learning and data mining techniques have not yet been widely used, probably because of the lack of large and quality data sets. Exercise, digital device use, places visited during the day and before bedtime, and sleep environment were the most identified factors associated with sleep quality. We identified key challenges surrounding the collection of high-quality sleep tracking data sets with consumer-grade sensors and in naturalistic settings as well as the extraction of hidden knowledge that could be translated into actionable insights and personalized behavior interventions. We highlight that future research should develop data analytics techniques and prediction models that properly handle the within-individual variation and between-individual variation in sleep tracking data sets. We hope that this scoping review could lay the groundwork for future research on ubiquitous and personal sleep tracking.

## Abbreviations

PRISMA: Preferred Reporting Items for Systematic Reviews and Meta-Analyses
RQ: research question
SE: sleep efficiency
SOL: sleep onset latency
